# A Randomized Controlled Trial of Chinese Patent Medicine Xiao'er Biantong Granules in the Treatment of Functional Constipation in Children

**DOI:** 10.1155/2018/4941505

**Published:** 2018-04-05

**Authors:** Qiu-han Cai, Rong Ma, Si-yuan Hu, Xin-min Li, Xi-xiong Xiang, Ying Ding, Ping He, Xin-min Han, Ke Chang, Wei Zhang, Zheng Xue, Xue-feng Wang, Cheng-liang Zhong, Na Yang

**Affiliations:** ^1^Tianjin University of TCM, Tianjin 300193, China; ^2^The First Affiliated Hospital of Tianjin University of TCM, Tianjin 300193, China; ^3^Hubei Provincial Hospital of TCM, Wuhan 430060, China; ^4^The First Affiliated Hospital of Henan University of TCM, Zhengzhou 136300, China; ^5^Yunnan Provincial Hospital of TCM, Kunming 650000, China; ^6^Jiangsu Province Hospital of TCM, Nanjing 210029, China; ^7^The Affiliated Hospital of Sichuan University of TCM, Chengdu 136300, China; ^8^The First Affiliated Hospital of Heilongjiang University of TCM, Harbin 150040, China; ^9^Shanghai Municipal Hospital of TCM, Shanghai 200071, China; ^10^The Affiliated Hospital of Liaoning University of TCM, Shenyang 110167, China

## Abstract

**Objective:**

To confirm the effect and safety of Xiao'er Biantong (XEBT) granules for treating chronic constipation in children.

**Methods:**

This randomized, double-blind, multicenter study enrolled 480 children with age of 1–14 years who had FC. All of them were randomly assigned to receive either XEBT granules or its placebo in the ratio of 3 : 1. The primary efficacy outcome was the frequency of spontaneous bowel movements (SBM) for 14 days, and secondary outcomes were effectual time, score of main symptoms, effect of constipation, disappearance rate of accompanying symptoms, and recurrence rate. We also observed the adverse event (AE) and adverse drug reaction (ADR) to evaluate safety.

**Results:**

The sociodemographic characteristics and efficiency data were comparable in the two groups at baseline. The mean values of SBM for 14 days were 8.89 and 5.63 in the XEBT group and the placebo group, respectively, and there were 86.87% and 30.91% subjects in two groups up to SBM ≥ 3/week, respectively. There were significant differences between the two groups. The effects in the XEBT group on median effectual time of defecation, main symptom score, disappearance rate of symptoms, and the differences were significant. The conclusions based on full analysis set (FAS) and per protocol set (PPS) were consistent. Nine AEs were reported, of which 7 were in the XEBT group (2.02%) while 2 were in the placebo group (1.77%). There were no significant differences in the occurrence rate of AE and ADR between the two groups.

**Conclusions:**

Xiao'er Biantong granules have superior efficacy compared to the placebo for the treatment of functional constipation in children and are well tolerated.

## 1. Introduction

Functional constipation is a widespread common chronic disorder in children. Its average morbidity is 14% and tends to increase with age. Approximately 2.9% of infants less than 1 year old have FC, while the rate increases to 10% in children aged 1-2 years [1]. It is considered to have relations with gender, family history, water intake, siblings, feeding patterns, age of toilet training, obesity, and so on [2, 3]. Typical symptoms include infrequent defecation, dry and hard stools, and encopresis, with accompanying symptoms, such as abdominal pain, decreased appetite, postprandial bloating, and even urine storage and urinary tract infection. Approximately 50~70% of all children with functional constipation recover and are taken off medication within 6 to 12 months [4, 5], but about one-fourth continue to experience symptoms in adulthood [6]. Constipation rarely has fatal complications, but has a significant impact on ultimately impairing health related quality of life for children and their families [7, 8]. A study found that children with FC are more vulnerable to anxiety and depression compared with healthy children and even children with asthma [9]. FC poses a huge financial burden on healthcare, with an annual cost of 3.9 billion in diagnosis and treatment, as evaluated by the Medical Expenditure Panel Survey (MEPS) [7]. The medical treatment of functional constipation in children often consists of two steps: disimpaction treatment and maintenance treatment. Oral mineral oil and polyethylene glycol electrolyte solution are widely used as the initial step to solve rectal disimpaction. Maintenance treatment medicine can be categorized into osmotic laxatives, fecal softeners, stimulant laxatives, and rectal laxatives [10]. Laxatives in clinical practice are widely accepted, we found that they can be tolerated by patients, and constipation can recur easily. Though there is a lot of evidence showing that acupuncture is effective [11, 12], children cannot accept it easily for pain, and it is a hard try to persuade them to stay in one position for a long time. In China, more and more parents prefer to seek help from Chinese medicine. Chinese herbs are effective for treating constipation during clinical practice, and to facilitate carrying and taking the herb, increasing amounts of Chinese patent medicine have been developed in recent years. Xiao'er Biantong is the first patent medicine for treating functional constipation in children. As a new drug, there is little evidence supporting its efficacy and safety, and thus, we designed this clinical study. It is reported as follows.

## 2. Methods

### 2.1. Design Overview

This study was designed as a prospective, randomized, double-blind, and placebo controlled study. The study was registered in the Chinese Clinical Trial Registry (http://www.chictr.org.cn) under the study identifier ChiCTR-TRC-13003326. Protocols followed the Good Clinical Practice (GCP) guidelines and the Declaration of Helsinki and were approved by the ethics committees of each institution.

### 2.2. Setting and Participants

To avoid selective bias, objectives were collected from 2013 to 2015 in 7 medical centers in China in different provinces and had geographical representation. All patients or their statutory guardians read and signed the informed consent form (ICF) before entering the study.

Inclusion criteria: the study included children aged between 1 and 14 years who were diagnosed with functional constipation based on a modification of Rome IV criteria [1] for infants and preschool children and classified with food retention syndrome in traditional Chinese medicine [13]. Retention syndrome may have the following symptoms: (1) elongation of defecation time; (2) dry stool; (3) difficult defecation; (4) encopresis; (5) abdominal distention; (6) decreased appetite; (7) dry mouth; (8) halitosis; (9) feverish feeling in palms and soles; and (10) reddened tongue, yellowish and greasy coating, and slippery and forceful pulse. Diagnosis was confirmed when patients satisfied criteria (1) and (2) with at least 3 terms of (5)–(9) after evaluating the tongue and pulse.

Exclusion criteria: intestinal stenosis caused by rectum or colon disease, such as tumor, inflammation, anal fissure, Crohn's disease, congenital megacolon, intestinal obstruction, intestinal adhesions, colonic polyps, and intestinal tuberculosis; constipation caused by psychiatric disorders, mental disorders, endocrine disease, or other system disease; outlet obstructive constipation or mixed constipation; allergic constitution or known allergy to any component in the XEBT granules; presenting with cardiovascular, cerebrovascular, liver, kidney and hematopoietic system diseases, and other serious diseases; positive fecal occult blood; and medical history of FC in the past 2 weeks.

### 2.3. Sample Size

Based on few published poststudies on XEBT granules, we followed the stipulation of the Provisions for Drug Registration published by the CFDA in 2007 and determined the sample size as 480. To ensure the statistical significance and consider the research grant, we randomized the objectives into two groups in a 3 : 1 ratio (3 for the XEBT group and 1 for the placebo group).

### 2.4. Randomization and Intervention

We used the stratified block randomization method with centers as the key stratification factor. A randomized list was made by SAS9.13, enclosed in an opaque envelop, and kept confidential in a safe location. We also made a single emergency envelope for every subject, with information on subject ID, drug group, and treatment measure for AE.

A double-blind method was used in this study. We blinded the random result twice, named the drugs as drug A and drug B instead of the real name in the first level, and named the groups as group 1 and group 2 instead of treatment or placebo group in the second level. The second level could be unblinded for analysis, while the first level should be unblinded until trial summary. The single emergency envelope can be opened under the following conditions: the occurrence of a serious adverse event (SAE), the occurrence of serious complications, or disease progression and need for necessary emergency treatments.

Eligible subjects were randomly assigned to receive either XEBT or placebo for 14 days. The placebo was similar in size, color, smell, taste, and appearance and had the same usage and dosage as XEBT. For children 1–3 y, the dosage was 2.5 g per time and 3 times a day; for children 4–6 y, the dosage was 5 g per time and 2 times a day; and for children > 7 y, the dosage was 5 g per time and 3 times a day. For observing SBM, the very first time taking the drugs should be after defecation. Other laxatives or treatment was forbidden during the study, including Chinese patent medicine, herbs, Western medicine, acupuncture, and Tuina massage. Parents were permitted to give a glycerine enema if the child failed to produce stool for 5 days. It should be recorded in detail in the case report form and is treated as a noneffective case.

### 2.5. Outcomes and Measurements

#### 2.5.1. Primary Efficiency Outcome

Frequency of spontaneous bowel movements (SBM) for 14 days is the primary efficacy measure. The data were collected from a bowel diary filled out by parents.

#### 2.5.2. Secondary Efficiency Outcomes

*(1) Effectual Time of Defecation (Day).* Effectual time means the interval time from the day of taking the drug to the first bowel movement. 

*(2) Main Symptom Scores.* See Table 1.

*(3) Disappearance Rate of Symptoms.* The main symptoms include defecation interval time, dry stool, difficult defecation, and encopresis. Accompanying symptom refers to abdominal distension, decreased appetite, dry mouth, halitosis, feverish feeling in palms and soles, and hyperchromic urine. We evaluated and recorded the score of symptoms on baseline and on the 14th day.

*(4) Recurrence Rate.* Subjects who were clinically cured in 14 days had a follow-up visit 2 weeks later to determine whether the constipation occurred again. Clinically cured was defined as a total score of main symptoms that decreased at least 90% compared to baseline.

### 2.6. Safety Outcomes

Occurrence rate of adverse events (AEs) and serious adverse events (SAE) is the primary safety outcome measure. We also performed a laboratory examination and evaluated vital signs, including breath, heart rate, blood pressure, temperature, ECG, routine blood and urine examination, and hepatic and renal function.

### 2.7. Statistical Analysis

Randomized subjects were assessed by comparing baseline characteristics between the two groups, using an unpaired *T*-test for continuous variables and a chi-squared test or a Wilcoxon rank-sum test for categorical variables. Analysis of covariance (ANCOVA) was used for taking the covariate into account, while CMH *χ*^2^ was used for considering the centers' influence. Missing data caused by off cases were imputed according to the principle of the last observation carried forward (LOCF). All hypothesis testing adopted a two-sided test, with a significance level of 0.05 between groups. Efficiency measure data were analyzed based on FAS and PPS while safety measures were analyzed in the safety set (SS). The superiority test on primary efficiency had an outcome measure of △ = 15%.

## 3. Results

### 3.1. Participant Characteristics

**Figure 1:**
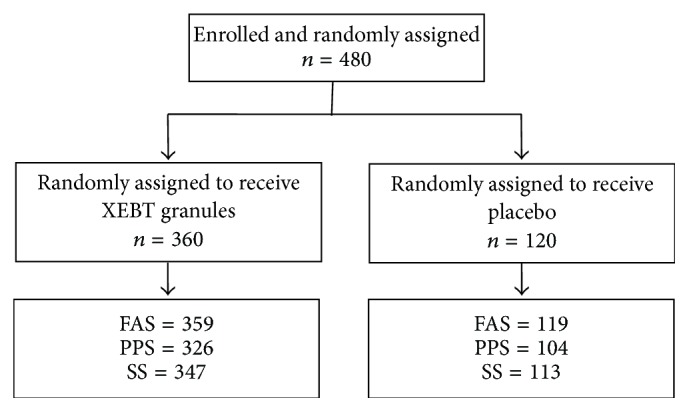
Patient flow through the study.

A total of 480 subjects were enrolled in this study, of which 360 were in the XEBT group and 120 were in the placebo group. One case satisfied the exclusion criteria, and one patient refused to take medicine, resulting in 478 cases constituting FAS. Forty-eight cases in FAS were excluded from the PPS due to major protocol violations and poor medicine compliance, and 460 cases had a complete safety record and were entered into the safety set. The flow of patients thorough the study is illustrated in Figure 1.

The sociodemographic characteristics and efficiency data were comparable in the two groups at baseline. The results are shown in Tables 2 and 3. There was no significant difference between the two groups in the course of disease, disease history, allergy history, medicine history, and main and accompanying symptom score. The baseline is comparable.

### 3.2. SBM

The mean values of SBM for 14 days in FAS (PPS) were 8.89 (8.93) and 5.63 (5.56) in the XEBT group and the placebo group, respectively. Considering the defecation interval time of the baseline as a concomitant variable, the Lsmean of SBM in FAS (PPS) was 8.88 (8.91) and 5.70 (5.64). There were significant differences between the two groups. See Table 4.

SBM ≥ 3/week was taken as the standard of normal defecating frequency according to Rome IV. The standardized rates were 86.87% (87.42%) and 30.91% (29.13%) in the XEBT group and the placebo group, respectively, and the difference had statistical significance. The difference between the two groups was 55.96% (46.4%, 65.52%), and the result of the superiority test implied that the difference had clinical significance. The conclusions based on FAS and PPS were consistent. See Table 5.

### 3.3. Effectual Time of Defecation (Day)

The median effectual time of defecation in FAS (PPS) was 1 (1) day and 2 (2) days in the XEBT group and the placebo group, respectively. There was a significant difference (56.1613, *P* = 0.0001) between the two groups. The conclusions based on FAS and PPS were consistent.

### 3.4. Main Symptom Score

The mean score of the main symptoms of FAS (PPS) was 2.08 (1.61) and 7.89 (7.65) in the XEBT group and the placebo group, respectively, compared with the score at baseline, and the mean decreased values were 8.76 (9.22) and 3.00 (3.19). There were significant differences between the two groups. The conclusions based on FAS and PPS were consistent. See Table 6.

### 3.5. Disappearance Rate of Symptoms

The disappearance rate was higher in the XEBT group than in the placebo group on symptoms of long defecation internal time, dry stool, difficult defecation, abdominal distension, decreased appetite, dry mouth, halitosis, feverish feeling in palms and soles, and hyperchromic urine. There were significant differences between the two groups. The encopresis disappearance rate was similar in two groups, but the sample size was too small to confirm the results. The conclusions based on FAS and PPS were consistent. See Table 7.

### 3.6. Recurrence Rate

There were 199 subjects satisfying the standards for clinical cure when the study concluded, including 195 cases (54.32%) in the XEBT group and 4 cases (3.36%) in the placebo group. After a 2-week follow-up visit, there were 25 cases who relapsed in the XEBT group and 1 case in the placebo group. The recurrence rate was higher in the placebo group, but there were no significant differences between them. The conclusions based on FAS and PPS were consistent. See Table 8.

### 3.7. Medication Compliance and Drug Combination

The medication compliance was 94.03 ± 13.86 and 92.28 ± 13.92 in the XEBT and placebo groups, respectively. There were 2 subjects in the XEBT group (0.58%) and 2 subjects (1.77%) in the placebo group who had drug combination. There were no significant differences.

Thus, this finding implies that medication compliance and drug combination are not interference factors for the results of this study.

### 3.8. Safety

Nine adverse events (AEs) were reported, 7 of which were in the XEBT group (2.02%) while 2 were in the placebo group (1.77%). Five AEs were recognized as an adverse drug reaction (ADR) by investigators, 4 of which were in the XEBT group (1.15%) and 1 was in the placebo group (0.88%). There were no significant differences in the occurrence rate of AE and ADR between the two groups. The ADR clinical manifestations included loose stool (1 case), diarrhea (3 cases), and vomit (1 case), all of which had slight symptoms with favorable prognosis. These ADRs can be attributed to the working mechanism of the drug. There was no significant difference in the variation of laboratory examinations between the two groups, except for creatinine, temperature, and heart rate. However, changes in those values were within the normal range and evaluated as being without clinical significance by investigators.

## 4. Discussion

Xiao'er Biantong granules are a Chinese patent medicine composed of 7 herbs. Pharmacodynamics studies showed that they can promote small bowel peristalsis and work against atropine-induced small intestine suppression, leading to increased gastric emptying movement and intestinal transit rate, as well as shortened defecation interval time in mice [14]. Houpo can regulate GI motility through various regulatory factors, such as ACH and serotonin (5-hydroxytryptamine, 5-HT) to solve abdominal bloating, constipation, and abdominal pain [15]. In recent years, an increasing number of studies have shown that FC is related to brain function abnormality and mental disorders. Houpo, which contains magnolol, is also used to treat clinical depression and anxiety-related disorders for its anxiolytic effect [16, 17] and thus may possibly treat FC. JueMingZi, which contains anthraquinones [18], could improve motor function in mice colon, reduce expression of AQP3 in colon mucosa, and have significant effects on the treatment of slow-transit constipation [19]. LuHui can be metabolized by the colonic flora to reactive Aloe-emodin, which is responsible for the purgative activity [20]. It had slight improvement in gastrointestinal peristalsis and reduction of fluid reabsorption and soft stool by stimulating the cholinergic receptor or competitively antagonizing with the u-opioid receptor on the intestinal wall [21]. BaiZhu contains* Atractylodes japonica*; it may specifically act on the distal colon longitudinal muscles among gastrointestinal smooth muscle [22]. Pharmacodynamics studies can explain the mechanism on the basis of modern medicine, while traditional Chinese medicine has a different view on this matter.

Traditional Chinese medicine holds that the spleen and stomach are the most important organs for digestion. The stomach receives food and drink, while the spleen governs transportation and transformation of food essence and fluid as the root of acquired constitution. Physiological and pathological characteristics of children are different compared to adults, due to their immature digestive organs with insufficient function. Improper feeding increases the burden of the stomach and intestine, leading to food stagnation. This disturbs qi movement so that the weakened qi cannot push the chime to move powerfully and quickly in the intestine. Based on the above mechanism of food retention syndrome, the principle of treatment should be to remove food retention (Houpo, LaiFuZi), promote defecation (XingRen, LuHui, and JueMingZi,), regulate qi movement (HouPo, ZhiQiao), and strengthen and nourish the spleen and stomach (BaiZhu). The component of Xiao'er Biantong granules is in accordance with TCM theoretical characteristics.

## 5. Conclusions

The result of this study supported that Xiao'er Biantong granules treatment of FC is rapid and can increase the frequency of defecation and shorten defecation interval time while addressing symptoms of dry stool, difficult defecation, encopresis, abdominal distension, decreased appetite, dry mouth, halitosis, hyperchromic urine, and feverish feeling in palms and soles. It is safe in clinical usage; ADRs usually appear as mild gastrointestinal symptoms and can be reversed quickly after drug withdrawal. In conclusion, Xiao'er Biantong granules have prospective clinical applications.

## Figures and Tables

**Table 1:** 

Score	0	2	4	6
Defecation interval time	1-2 days	3 days	4 days	≥5 days
Dry stool (Bristol stool scale)	Types 4–7	Type 3	Type 2	Type 1
Difficult defecation	Smooth bowel movement	With little difficultly	Needing whole body forced	Needing mannitol to help

Score	0		1	

Encopresis	Yes		No	

**Table 2:** Sociodemographic characteristics at baseline (*n*, %).

Terms	XEBT (*n* = 359)	Placebo (*n* = 119)	*P* value
Gender, *n* (%)			
Male	167 (46.52%)	60 (50.42%)	0.4601
Female	192 (53.48%)	59 (49.58)	
Age, years			
Mean ± SD	5.54 ± 3.07	5.69 ± 3.02	0.6643
Median [range]	4.83	5	
Height (cm)	112.35 ± 21.56	114.04 ± 21.86	0.4592
Weight (kg)	21.47 ± 9.57	22.78 ± 10.76	0.2254
Nation, *n* (%)			
Han nationality	350 (97.49%)	119 (100%)	0.1206
Other nationality	9 (2.51%)	0 (0%)	
Duration of FC (years)			
Mean ± SD	1.69 ± 1.78	1.68 ± 1.57	0.9466
Median [range]	1	1.08	

**Table 3:** Symptom scores at baseline (*n*, %).

Symptoms	Score	XEBT (*n* = 359)	Placebo (*n* = 119)	*P* value
Defecation interval time	2	195 (54.32%)	66 (55.46%)	0.7641
	4	143 (39.83%)	39 (32.77%)	
	6	21 (5.85%)	14 (11.76%)	
Dry stool	2	90 (25.07%)	35 (29.41%)	0.4353
	4	137 (38.16%)	43 (36.13%)	
	6	132 (36.77%)	41 (34.45%)	
Difficult defecation	0	4 (1.11%)	0 (0.00%)	0.559
	2	119 (33.15%)	38 (31.93%)	
	4	195 (54.32%)	66 (55.46%)	
	6	41 (11.42%)	15 (12.61%)	
Encopresis	0	355 (98.89%)	118 (99.16%)	1
	1	4 (1.11%)	1 (0.84%)	
Abdominal distension	0	54 (15.04%)	21 (17.65%)	0.9499
	1	171 (47.63%)	50 (42.02%)	
	2	117 (32.59%)	46 (38.66%)	
	3	17 (4.74%)	2 (1.68%)	
Decreased appetite	0	116 (32.31%)	28 (23.53%)	0.0703
	1	243 (67.69%)	91 (76.47%)	
Dry mouth	0	124 (34.54%)	41 (34.45%)	0.9863
	1	235 (65.46%)	78 (65.55%)	
Halitosis	0	107 (29.81%)	35 (29.41%)	0.9352
	1	252 (70.19%)	84 (70.59%)	
Feverish feeling in palms and soles	0	134 (37.33%)	46 (38.66%)	0.7953
	1	225 (62.67%)	73 (61.34%)	
Hyperchromic urine	0	121 (33.70%)	51 (42.86%)	0.0714
	1	238 (66.30%)	68 (57.14%)	
Score of four main symptoms	Mean ± SD	10.85 ± 2.84	10.89 ± 3.27	0.9021

**Table 4:** Spontaneous bowel movements (mean ± SD).

Group	*n*	1–7 days	7–14 days	1–14 days	*P* value
XEBT group	335	4.12 ± 1.61	4.77 ± 1.40	8.89 ± 2.75	0.0001
Placebo group	110	2.72 ± 1.29	2.91 ± 1.29	5.63 ± 2.41	

**Table 5:** Recovery rate for SBM (*n*, %).

Group	*n*	SBM ≥ 3/w	SBM < 3/w	*P* value	Difference	95% CI
XEBT group	335	291 (86.87%)	44 (13.13%)	0.0001	55.96%	46.4%, 65.52%
Placebo group	110	34 (30.91%)	76 (69.09%)			

**Table 6:** Main symptom scores (mean ± SD).

Group	*n*	Baseline	14th day	Decreased value	Decreased rate
XEBT	335	10.85 ± 2.84	2.08 ± 3.02	8.76 ± 3.92	80.01 ± 28.78
Placebo	110	10.89 ± 3.27	7.89 ± 3.51	3.00 ± 3.26	25.79 ± 28.89
*P* value		0.902	0.0001	0.0001	0.0001

**Table 7:** Disappearance rate of symptoms (*n*, %).

Symptoms	XEBT group		Placebo group		*P* value
	*n*	Disappeared	*n*	Disappeared	
Long defecation interval time	359	299 (83.29%)	119	22 (18.49%)	0.0001
Dry stool	359	236 (65.74%)	119	11 (9.24%)	0.0001
Difficult defecation	356	238 (66.85%)	119	11 (9.24%)	0.0001
Encopresis	5	3 (60.00%)	3	0 (0.00%)	0.1964
Abdominal distension	309	214 (69.26%)	101	23 (22.77%)	0.0001
Decreased appetite	255	181 (70.98%)	94	29 (30.85%)	0.0001
Dry mouth	248	166 (66.94%)	81	31 (38.27%)	0.0001
Halitosis	260	185 (71.15%)	90	35 (38.89%)	0.0001
Feverish feeling in palms and soles	235	152 (64.68%)	80	24 (30.00%)	0.0001
Hyperchromic urine	246	193 (78.46%)	77	24 (31.17%)	0.0001

**Table 8:** Recurrence rate (*n*, %).

Group	*n*	Relapsed	Healthy	*P* value
XEBT group	195	25 (12.82%)	170 (87.18%)	0.4314
Placebo group	4	1 (25.00%)	3 (75.00%)	

## References

[1] Drossman D. A., Hasler W. L. (2016). Rome IV—functional GI disorders: disorders of gut-brain interaction. *Gastroenterology*.

[2] Benninga M. A., Voskuijl W. P., Taminiau J. A. J. M. (2004). Childhood constipation: Is there new light in the tunnel?. *Journal of Pediatric Gastroenterology and Nutrition*.

[3] Loening-Baucke V. (2007). Prevalence rates for constipation and faecal and urinary incontinence. *Archives of Disease in Childhood*.

[4] Pijpers M. A. M., Bongers M. E. J., Benninga M. A., Berger M. Y. (2010). Functional constipation in children: A systematic review on prognosis and predictive factors. *Journal of Pediatric Gastroenterology and Nutrition*.

[5] Biggs W.-S., Dery W.-H. (2006). Evaluation and treatment of constipation in infants and children. *American Family Physician*.

[6] Bongers M. E. J., Van Wijk M. P., Reitsma J. B., Benninga M. A. (2010). Long-term prognosis for childhood constipation: Clinical outcomes in adulthood. *Pediatrics*.

[7] Liem O., Harman J., Benninga M., Kelleher K., Mousa H., Di Lorenzo C. (2009). Health utilization and cost impact of childhood constipation in the United States. *Journal of Pediatrics*.

[8] Wang C., Shang L., Zhang Y. (2013). Impact of functional constipation on health-related quality of life in preschool children and their families in xi’an, China. *PLoS ONE*.

[9] Olaru C., Diaconescu S., Trandafir L. (2016). Chronic Functional Constipation and Encopresis in Children in Relationship with the Psychosocial Environment. *Gastroenterology Research and Practice*.

[10] Hoekman D. R., Benninga M. A. (2013). Functional constipation in childhood: Current pharmacotherapy and future perspectives. *Expert Opinion on Pharmacotherapy*.

[11] Zheng Q., Zheng H., Lu L. (2015). Acupuncture for functional constipation: protocol of an individual patient data meta-analysis. *BMJ Open*.

[12] Tjen-A-Looi S. C., Fu L. (2017). Sustained effects of acupuncture in treatment of chronic constipation. *Annals of Palliative Medicine*.

[13] State Administration of Traditional Chinese Medicine of the People's Republic of China *Standardization of Diagnosis And Curative Effect on Chinese Syndromes*.

[14] Dong Z.-B., Huang S.-F., XU P.-F. (2006). Study on the Diarrhea and Purgative Function of Xiao`er Bian Tong Granules. *An Hui Medical and Pharmaceutical Journal*.

[15] Poivre M., Duez P. (2017). Biological activity and toxicity of the Chinese herb Magnolia officinalis Rehder & E. Wilson (Houpo) and its constituents. *Journal of Zhejiang University SCIENCE B*.

[16] Kuribara H., Stavinoha W. B., Maruyama Y. (1998). Behavioural pharmacological characteristics of honokiol, an anxiolytic agent present in extracts of Magnolia bark, evaluated by an elevated plus-maze test in mice. *Journal of Pharmacy and Pharmacology*.

[17] Kuribara H., Kishi E., Hattori N., Yuzurihara M., Maruyama Y. (1999). Application of the elevated plus-maze test in mice for evaluation of the content of honokiol in water extracts of magnolia. *Phytotherapy Research*.

[18] Yang C., Wang S., Guo X., Sun J., Liu L., Wu L. (2015). Simultaneous determination of seven anthraquinones in rat plasma by Ultra High Performance Liquid Chromatography-tandem Mass Spectrometry and pharmacokinetic study after oral administration of Semen Cassiae extract. *Journal of Ethnopharmacology*.

[19] Liu X., Du A.-L., Jiang H.-B. (2015). Effects of Semen Cassiae on Colonic Myoelectrical Activity and AQP3 in Mice. *Chinese Journal of Gerontology*.

[20] Radha M. H., Laxmipriya N. P. (2015). Evaluation of biological properties and clinical effectiveness of *Aloe vera*: a systematic review. *Journal of Traditional and Complementary Medicine*.

[21] Lin Z.-H., Wu J.-X., Xiao ZD Z.-D. (2005). The curative effect of aloe on constipation and its primary mechanism. *Guangdong Medical Journal*.

[22] Choi K. H., Jeong S. I., Lee J. H. (2011). Pharmacological mechanism responsible for the Atractylodes japonica-induced distal colonic contraction in rats. *Phytomedicine*.

