# Corrigendum to “A Randomized Controlled Trial of Chinese Patent Medicine Xiao'er Biantong Granules in the Treatment of Functional Constipation in Children”

**DOI:** 10.1155/2018/3616345

**Published:** 2018-12-02

**Authors:** Qiu-han Cai, Rong Ma, Si-yuan Hu, Xin-min Li, Xi-xiong Xiang, Ying Ding, Ping He, Xin-min Han, Ke Chang, Wei Zhang, Zheng Xue, Xue-feng Wang, Cheng-liang Zhong, Na Yang

**Affiliations:** ^1^Tianjin University of TCM, Tianjin 300193, China; ^2^First Teaching Hospital of Tianjin University of Traditional Chinese Medicine, Tianjin 300193, China; ^3^Hubei Provincial Hospital of TCM, Wuhan 430060, China; ^4^The First Affiliated Hospital of Henan University of TCM, Zhengzhou 136300, China; ^5^Yunnan Provincial Hospital of TCM, Kunming 650000, China; ^6^Jiangsu Province Hospital of TCM, Nanjing 210029, China; ^7^The Affiliated Hospital of Sichuan University of TCM, Chengdu 136300, China; ^8^The First Affiliated Hospital of Heilongjiang University of TCM, Harbin 150040, China; ^9^Shanghai Municipal Hospital of TCM, Shanghai 200071, China; ^10^The Affiliated Hospital of Liaoning University of TCM, Shenyang 110167, China

In the article titled “A Randomized Controlled Trial of Chinese Patent Medicine Xiao'er Biantong Granules in the Treatment of Functional Constipation in Children [1]”, there was an error in the second affiliation. The corrected affiliations are shown above.
